# Medicine in Fiction

**Published:** 1895-08

**Authors:** 


### MEDICINE IN FICTION.

Nearly a decade ago, the *Medical Standard* inaugurated a discussion of medicine in fiction which has since spread across the Atlantic. It is a singular fact that many criticisms on medicine in fiction display less knowledge of medicine than the author criticised. This has been noticeably the case with the *British Medical Journal.* Dr. Conan Doyle has introduced a chapter in his "Round the Red Lamp" discussing this subject through the medium of physicians exchanging experiences.

Doyle makes one of these remarks, evidently in total ignorance of the literature on the subject: "There is no need for fiction in medicine, for the facts will always beat anything you can fancy. But it has seemed to me sometimes that a curious paper might be read about the use of medicine in popular fiction; of what the folk die and what diseases are made most use of in novels. Some are worn to pieces and others, which are equally common in real life, are never mentioned. Typhoid is fairly frequent, but scarlet fever is unknown. Heart disease is common, but then heart disease as we know it is usually the sequel of some foregoing disease of which we never hear anything in the romance. Then there is the mysterious malady called brain fever, which always attacks the heroine after a crisis, but which is unknown under that name to the textbook. People, when they are over-excited in novels, fall down in a fit. In fairly large experience I have never known anyone do so in real life. The small complaints simply don't exist. Nobody ever gets shingles or quinsy or mumps in a novel. All the diseases, too, belong to the upper part of the body. The novelist never strikes below the belt."

Doyle seems singularly unacquainted with his two professions. Wilkie Collins uses scarlet fever in "Blind Love." "Brain fever" is a popular term for meningitis and is but too often applied to other delirious states by physicians. Over-excitement may cause syncopic states, apoplexy, and hysteria, all of which would be popularly called fits. Of course, except in larvated epileptics, true epilepsy is never thus produced. Larvated epilepsy is sufficiently obscure, both to the profession and the public, for its first motor expression to be regarded as a first fit. Emotional excitement notoriously brings on such attacks.—*Medical Standard.*

